# Factors affecting sufentanil consumption for intravenous controlled analgesia after hepatectomy: retrospective analysis

**DOI:** 10.1186/s12871-021-01526-z

**Published:** 2021-12-07

**Authors:** Yue Wu, Lina Tian, Chunye Li, Minjun Liu, Shina Qiao, Weibo Zhang, Suming Tian, Gang Chen

**Affiliations:** 1 Department of Anesthesiology, Sir Run Run Shaw Hospital, School of Medicine, Zhejang University, 3 Qingchun Road East, ShangCheng District, Hangzhou 310016 Zhejiang, People’s Republic of China; 2 Department of Pain, Affiliated Hospital of Jiangsu University, 438 Jiefang Road, Zhenjiang, 212001 Jiangsu People’s Republic of China; 3 Department of Nursing Education, Sir Run Run Shaw Hospital, School of Medicine, Zhejang University, 3 Qingchun Road East, ShangCheng District, Hangzhou 310016 Zhejiang, People’s Republic of China

**Keywords:** Influencing factors, Postoperative pain, Patient-controlled analgesia, Sufentanil consumption, Hepatectomy

## Abstract

**Background:**

Pain control after hepatectomy is usually achieved by opioids. There are significant individual differences in the amount of opioids used after hepatectomy, and the metabolism of opioids is liver-dependent. The purpose of our study was to explore the possible risk factors for opioid consumption during the first 48 h after surgery.

**Methods:**

In a retrospective study design involving 562 patients undergoing open or laparoscopic hepatectomy, all patients were treated with intravenous patient-controlled analgesia (IV-PCA) along with continuous and bolus doses of sufentanil for a duration of 48 h after surgery during the time period of August 2015 and February 2019. The primary endpoint was high sufentanil consumption 48 h after hepatectomy, and patients were divided into two groups: those with or without a high PCA sufentanil dosage depending on the third quartile (Q3). The secondary endpoint was the effect of a high PCA sufentanil dosage on various possible clinical risk factors. The relevant parameters were collected, and correlation and multivariate regression analyses were performed.

**Results:**

The median operation time was 185 min (range, 115–250 min), and the median consumption of sufentanil 48 h after the operation was 91 μg (IQR, 64.00, 133.00). Factors related to the consumption of sufentanil at 48 h after hepatectomy included age, operation time, blood loss, intraoperative infusion (red blood cells and fresh-frozen plasma), pain during movement after surgery (day 1 and day 2), preoperative albumin, and postoperative blood urea nitrogen. Age (≤ 60 and > 60 years), extent of resection (minor hepatic resection and major hepatic resection), surgical approach (laparoscope and open) and operation time (min) were independent risk factors for sufentanil consumption at 48 h postoperatively.

**Conclusion:**

Age younger than 60 years, major hepatic resection, an open approach and a longer operation are factors more likely to cause patients to require higher doses of sufentanil after hepatectomy, and the early identification of such patients can increase the efficacy of perioperative pain management.

## Background

Severe pain stress occurs after liver cancer surgery, which may lead to severe liver function damage, increase immune suppression, and promote postoperative infection, tumor recurrence and metastasis [1, 2]. Continuous and adequate analgesia can reduce postoperative pain, decrease postoperative complications, and improve postoperative patient satisfaction. Additionally, analgesia plays a crucial role in improving perioperative safety, for example, enhancing the protection of immune function, reducing postoperative infection and promoting the recovery of liver function [3–5]. Therefore, adequate postoperative analgesia is crucial to improve the prognosis of patients undergoing liver cancer surgery.

Laparoscopic hepatectomy has been widely used and carried out in hospitals worldwide. Although it is a minimally invasive surgery with a small incision, laparoscopic surgery can also cause severe visceral pain in deep tissues due to the unique anatomical structure of the liver and the unusually rich liver blood vessels. Moreover, carbon dioxide pneumoperitoneum stimulates the gut and somatic nerves [6], which can also aggravate surgical trauma and pain stress.

Therefore, pain not only after open hepatectomy but also after laparoscopic surgery should be of great concern. Multimode analgesia is an important concept and method of postoperative analgesia, which requires the combination of analgesic drugs with different mechanisms of action and different analgesic measures to achieve the best analgesic effect and minimize adverse reactions. Opioid drugs are indispensable in postoperative analgesia, and patient-controlled analgesia (PCA) is the most commonly used and ideal postoperative analgesia method, especially for abdominal surgery. An increasing number of studies have proven that sufentanil, as a commonly used opioid, has significant advantages in the safety and effectiveness of postoperative intravenous PCA (IV-PCA) [7]. However, there are substantial individual differences in opioid use. The characteristics of the patient, tumor, surgery, and even anesthesia may influence the amount of opioids used after surgery. The metabolism and elimination of opioids depends on liver and kidney function. Other studies have shown that preoperative platelet and coagulation abnormalities are independent risk factors for significant postoperative mortality in patients with liver cancer [8] and may also have an impact on postoperative opioid use. Therefore, the individualized postoperative PCA scheme can help to reduce postoperative pain stress, improve patient satisfaction, and reduce the waste of workforce and material resources.

The early identification of risk factors for postoperative opioid use and increased awareness of the importance of related risk factors will contribute to more effective intervention and better pain management. In this study, patients with primary liver cancer who underwent laparoscopic or open hepatectomy and received IV-PCA after surgery were selected as the study subjects. We retrospectively analyzed the influencing factors of the postoperative sufentanil dosage to provide a basis for individualized postoperative analgesic treatment.

## Methods

The Institutional Review Board of Sir Run Run Shaw Hospital of Zhejiang University, Hangzhou, Zhejiang, P.R. China approved the study protocol. A retrospective review of all patients undergoing hepatectomy at a single institution between August 2015 and February 2019 was performed. All patients with primary liver cancer who underwent hepatectomy were included in the study. All cases were confirmed to be hepatocellular carcinoma by postoperative histopathology. The exclusion criteria were (1) a history of chronic pain and mental illness, (2) the use of analgesics before surgery, (3) transfer to the intensive care unit (ICU) after surgery, and (4) the use of opioids other than sufentanil postoperatively.

Types of hepatic resection were defined by consensus [9]. Right hepatectomy, left hepatectomy, extended right hepatectomy, and extended left hepatectomy were considered major hepatic resection (three segments or more), whereas segmentectomy of one or two segments and nonanatomic wedge resection were classified as minor hepatic resection (two segments or fewer).

All patients undergoing either open or laparoscopic hepatectomy received general anesthesia with endotracheal intubation. Anesthesia was induced by sufentanil (IDT Biologika GmbH, Ampharmapark D-06861 Dessau-Roblau, Germany), propofol (Fresenius Kabi Deutschland GmbH, Germany), benzocisuatracurium (Zhejiang Xianju Pharmaceutical Co., Ltd., China) or rocuronium bromide (Zhejiang Xianju Pharmaceutical Co., Ltd., China), and anesthesia was maintained by the continuous infusion of remifentanil (Yichang Renfu Pharmaceutical Co., Ltd., China) and inhalation of sevoflurane (Shanghai Hengrui Pharmaceutical Co., Ltd., China) and intermittent injection of benzocisuatracurium or rocuronium bromide.

According to the patient’s condition and the preference of the anesthesiologist, most patients were intravenously infused with dexmedetomidine (Yangzijiang Pharmaceutical Group Co., Ltd., China) during the operation. At the end of laparoscopic surgery, 10–15 ml of 0.375% ropivacaine (AstraZeneca AB, Britain) was routinely administered for incisional local infiltration analgesia.

The IV-PCA regimen typically consisted of sufentanil 250 μg plus normal saline (total volume of 250 ml). Sufentanil was administered through a pump, programmed to deliver 1 ml/hour as a background infusion, and 2 ml per demand. Postoperative pain at rest and during movement, such as coughing and turning over, was recorded and assessed using a numerical rating scale (NRS) [10] ranging from 0, indicating no pain, to 10, indicating severe pain. The patient selected the corresponding number to indicate the degree of pain, with an NRS score ≤ 3 serving as the control target. Preoperative patients routinely received education on how to use the PCA pump. If the postoperative NRS score was ≥4, they could give the required amount (bolus dose) by themselves through the button until they reached an NRS score ≤ 3. The nurse in charge performed a routine pain assessment once every 4 h; an intervention and reassessment were given when the NRS score was ≥4, and routine assessment was resumed when the NRS score was ≤3. In addition, the Acute Pain Service Team (APS) conducted postoperative follow-up examinations twice a day for patients who used a PCA pump after hepatectomy to observe and record the patient’s pain score and adverse reactions, such as pain at rest or during movement that could not be effectively controlled or medication overdoses causing drowsiness and even respiratory depression, to adjust the analgesic pump parameters in a timely manner. At other times, if the doctor in charge or nurse reported the patient’s pain or adverse reaction in a timely manner, the APS team addressed it as soon as possible.

In addition to sufentanil, many patients received a routine intravenous injection of anti-inflammatory and analgesic drugs (nonsteroidal anti-inflammatory drugs (NSAIDs)), such as flurbiprofen (Beijing Tide Pharmaceutical Co., Ltd., China) or parecoxib (Pharmacia & Upjohn Company LLC, USA), in the first 48 h after surgery.

Patients were routinely transferred to the general ward after hepatectomy (which differed from practice at other hospitals) unless there were issues such as respiratory and circulatory instability, unusual surgical complexity, and excessive bleeding. Routine blood and biochemical tests were performed within 48 h after surgery. Postoperative nausea and vomiting (PONV) are usually treated with metoclopramide (Suicheng Pharmaceutical Co., Ltd., China) or ondansetron (Qilu Pharmaceutical Co., Ltd., China).

Patient demographics and clinical data from the medical records included sex, age, body mass index (BMI; weight (kg)/height (meters) squared), tumor factors (tumor size, tumor number, differentiation, vascular invasion and lymph node invasion), perioperative factors (American Society of Anesthesiologists (ASA) physical status, surgical approach, extent of resection, operation time, cut margin, intraoperative blood loss, intraoperative transfusion, intraoperative dexmedetomidine use, postoperative NSAID use, postoperative NRS score, postoperative sufentanil dosage and postoperative adverse reactions) and laboratory data (platelet count, prothrombin time, indicators of liver and kidney function).

The primary endpoint was the level of sufentanil consumption 48 h after hepatectomy and the third quartile (Q3, 133 μg). The patients were divided into two groups: those with or without a high PCA sufentanil dosage depending on this value. Other parameters were compared between groups to assess whether there were differences. The secondary endpoint was the effect of a high PCA sufentanil dosage on various possible clinical risk factors, for example, patient demographics and clinical data, including sex, age, BMI, tumor factors, perioperative factors and laboratory data.

### Statistical analysis

All continuous data with a normal distribution are described as the mean ± SD, and continuous data with a nonnormal distribution are described as the median and interquartile range. Normality was assessed using the Shapiro-Wilk test. Categorical variables are expressed as numbers and percentages. Univariate analysis was performed on the preoperative demographic data and perioperative general characteristics of patients undergoing liver resection, including age, sex, BMI, intraoperative blood loss, operating time, intraoperative transfusion, extent of resection, surgical approach, ASA classification, intraoperative DEX, postoperative NSAIDs, PONV, pain score, sufentanil dosage, laboratory data and tumor-related factors, and independent sample t-test or Mann-Whitney U test were used to compare the differences between the PCA-NH and PCA-H groups; and the independent sample t-test or Mann-Whitney U test was also used to compare the difference in sufentanil consumption in PCA for each category. For categorical parameters, chi-square analysis and Fisher’s exact test were used when appropriate. Correlation analysis between possible risk factors and sufentanil consumption was carried out. The related variables and the factors that may affect the postoperative sufentanil dosage obtained from the univariate analysis were included in the multivariate regression analysis to identify the risk factors for postoperative PCA sufentanil consumption. Data were considered statistically significant at a *P*-value of less than 0.05. All analyses were performed using SPSS Statistics software (Version 23.0, Chicago, IL, USA).

## Results

**Fig. 1:**
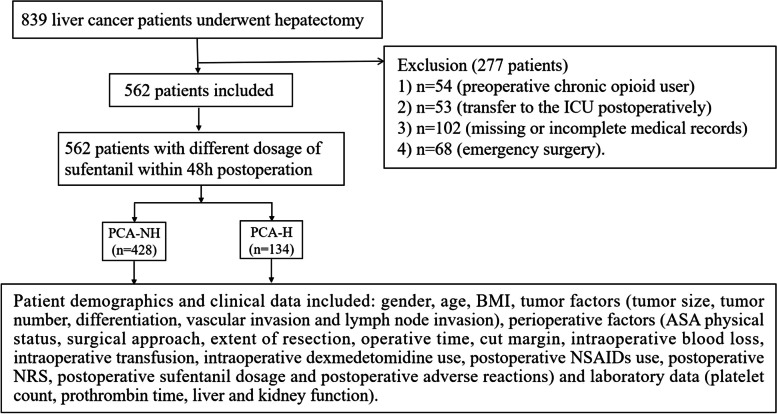
Patient selection flowchart

A total of 839 patients at the Sir Run Run Shaw Hospital underwent hepatectomy by a laparoscopic or open approach between August 2015 and February 2019. Of these, 54 were excluded due to preoperative chronic opioid use, 53 were excluded due to transfer to the ICU postoperatively, 102 were excluded due to missing or incomplete medical records, and 68 were excluded due to emergency surgery. As a result, 562 patients were included in the final analysis. A flowchart of the patient selection process is shown in Fig. 1.

### Patient characteristics and perioperative variables

**Fig. 2:**
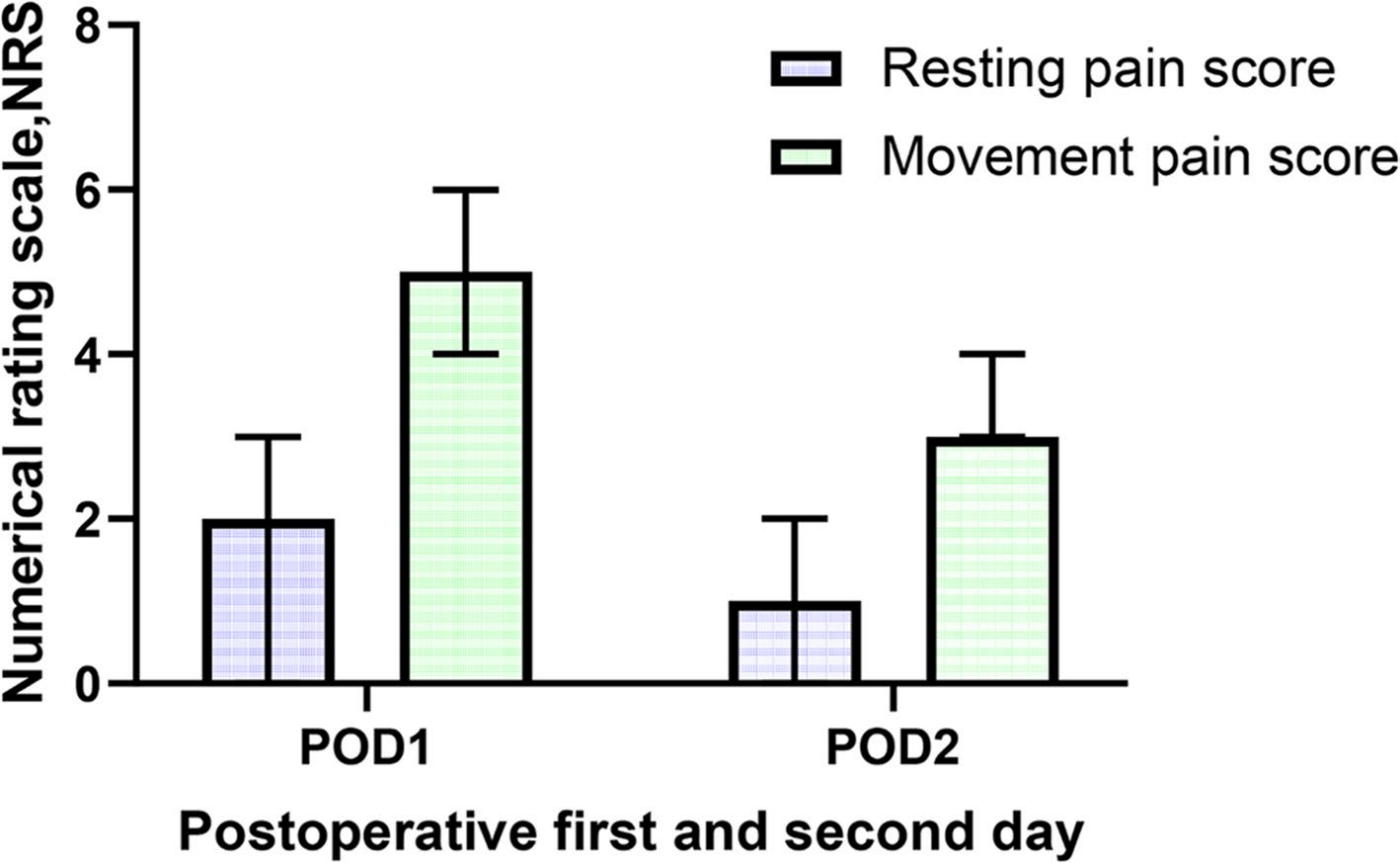
Postoperative pain score on day 1 and day 2 (median, IQR)

The preoperative demographic data and general perioperative characteristics of the total patient sample and the two groups of patients (with and without a high PCA sufentanil dosage, i.e., the PCA-NH and PCA-H groups) are presented in Table 1. Among the 562 participants, there were slightly more males (316, 56.2%) than females. The median age was 57.0 years (range, 18 ~ 88 ml), and the median BMI was 22.7 kg/m^2^ (range, 15.38 ~ 33.87 kg/m^2^). Pain during movement was significantly higher than pain at rest 2 days after the operation (*P* < 0.05), and the scores of pain at rest and during movement on the second day after the operation were significantly lower than those on the first day (*P* < 0.05) (Fig. 2). Thirty-seven patients (6.6%) had nausea and vomiting, 25 (4.4%) had dizziness, and 29 (5.2%) had drowsiness 48 h after hepatectomy, but no life-threatening opioid-related side effects (such as respiratory depression) were observed.

**Table 1:** The preoperative demographic data and general perioperative characteristics of patients undergoing hepatectomy

	All (*n* = 562)	PCA-NH (*n* = 428)		PCA-H (*n* = 134)		*P*-value
	Median (IQR)	n (%)	Median (IQR)	n (%)	Median (IQR)	
Age (years old)	57.00 (48.00, 65.00)		59.00 (49.00, 65.00)		53.78 (44.83, 61.58)	0.002*
Gender						
Male		230 (72.8)		86 (27.2)		0.034*
Female		198 (80.5)		48 (19.5)		
BMI (kg/m**2**)	22.7 (20.8, 22.3)		22.60 (20.67, 25.16)		23.35 (21.22, 25.30)	0.307
Intraoperative blood loss (ml)	200 (100, 500)		200.00 (100.00, 500.00)		292.31 (141.67, 714.29)	0.001*
Operative time (min)	210 (160, 300)		205 (156.25, 285.00)		245.38 (182.08, 320.00)	0.002*
Intraoperative transfusion						
No		289 (79.4)		75 (20.6)		0.015*
Yes		139 (70.2)		59 (29.8)		
RBC (u)	0 (0, 2.00)		0 (0, 1.50)		0.73 (0, 2.11)	0.164
FFP (ml)	0 (0, 352.50)		0 (0, 315.00)		1.42 (0, 385.71)	0.034*
Extent of resection						
Minor hepatic resection		246 (90.4)		26 (9.6)		< 0.001*
Major hepatic resection		182 (62.8)		108 (37.2)		
Surgical approach						
Laparoscope		251 (83.1)		51 (16.9)		< 0.001*
Open		177 (68.1)		83 (31.9)		
ASA classification						
I + II		386 (76.3)		120 (23.7)		0.831
III		42 (75.0)		14 (25.0)		
Introperative DEX						
No		52 (77.6)		15 (22.4)		0.766
Yes		376 (76.0)		119 (24.0)		
Postoperative NSAIDs						
No		301 (76.8)		91 (23.2)		0.595
Yes		127 (74.7)		43 (25.3)		
PONV						
No		404 (77.0)		121 (23.0)		0.095
Yes		24 (64.9)		13 (35.1)		
Resting pain score on POD1	2 (0, 3)		2 (0.25, 3)		2 (0,3)	0.251
Movement pain score on POD1	5 (4, 6)		5 (4, 6)		5 (5,6)	0.503
Resting pain score on POD2	1 (0, 2)		1 (0,2)		1 (0,2)	0.823
Movement pain score on POD2	3 (3, 4)		3 (3,4)		4 (3,4)	0.056
Sufentanil dosage 24 h (μg)	50 (34, 75)		42.00 (31.00, 56.75)		98.00 (80.00,118.00)	< 0.001*
Sufentanil dosage 48 h (μg)	91 (64, 133)		77.00 (60.00, 101.75)		173.00 (149.75, 205.75)	< 0.001*

*P value < 0.05 statistically significant, whether it is a high PCA sufentanil dose comparison between the two groups; Mann-Whitney U test; Chi-square test; n(%) = number (percentage); *PCA-H* High PCA sufentanil dosage, *PCA-NH* Non high PCA sufentanil dosage, *DEX* Dexmedetomidine, *NSAIDs* Anti-inflammatory and analgesic drugs, *RBC (u)* Intraoperative transfusion of red blood cells (unit), *FFP (ml)* Intraoperative transfusion of fresh frozen plasma (ml), *POD1* Postoperative first day, *POD2* Postoperative second day

There was no significant difference in BMI, intraoperative infusion of red blood cells (RBCs), ASA classification, intraoperative dexmedetomidine use, postoperative NSAID use, PONV, or pain scores 2 days after surgery. Compared with the PCA-NH group, the PCA-H group included younger patients, more males, more intraoperative transfusions, more major hepatic resections, and more open approaches (*P* < 0.05).

Table 2 shows the types of hepatic resection performed. Major hepatic resection was performed in 290 cases (51.6%), segmentectomy in 147 cases (26.2%), and localized resection in 125 cases (22.2%). All operations were mainly performed by open and laparoscopic approaches, including 260 cases of open surgery (46.3%) and 302 cases of laparoscopic surgery (53.7%), with a median surgical duration of 185 min (range, 115 ~ 250 min). The median intraoperative blood loss was 200 ml (range, 50 ~ 8000 ml), and intraoperative transfusion consisted mainly of RBCs and fresh-frozen plasma (FFP). Blood transfusion was required in 198 patients (35.2%), with a median of 0 U (range, 0 ~ 12.5 U) of RBCs and 0 ml (range, 0 ~ 3780 ml) of FFP (Table 1).

**Table 2:** Types of hepatic resection

Type of resection	N (%)
Major hepatic resection	290 (51.6)
Right lobectomy	159 (28.3)
Right hepatectomy	87 (15.5)
Left hepatectomy	44 (7.8)
Segmentectomy	147 (26.2)
Left lateral segmentectomy	75 (13.3)
Typical segmentectomy	72 (12.8)
Localized resection	125 (22.2)

The preoperative Child-Pugh grading of liver function in all patients was grade A. Table 3 shows the perioperative laboratory data of the total patient sample and the two groups of patients, including the platelet count, prothrombin time, and indicators of liver and kidney function (alanine aminotransferase, aspartate aminotransferase, alkaline phosphatase, albumin, urea nitrogen and creatinine). There were statistically significant differences in the preoperative BUN level, preoperative creatinine level, preoperative platelet count, and postoperative BUN level between the two groups. There were no significant differences in the other laboratory data between the two groups. The results are described in Table 3.

**Table 3:** Perioperative laboratory data of patients undergoing hepatectomy

	All (n = 562)	PCA-NH (n = 428)	PCA-H (n = 134)	***P***-value
	Median, IQR			
Preoperative laboratory data				
AST (U/l)	29.50 (21.00, 49.00)	30.00 (21.00, 52.75)	28.00 (21.00, 44.00)	0.204
ALT (U/l)	26.00 (16.00, 45.00)	27.00 (16.25, 46.00)	25.33 (15.38, 38.00)	0.179
Alkaline phosphatase (U/l)	109.00 (81.00, 181.50)	110.50 (83.00, 193.25)	106.33 (79.33, 164.00)	0.159
Albumin (g/l)	38.30 (34.70, 42.43)	38.20 (34.60, 42.18)	39.03 (35.10, 43.60)	0.139
BUN (mmol/l)	4.89 (3.89, 6.01)	4.79 (3.78, 5.87)	5.18 (4.12, 6.38)	0.008*
Creatinine (μmol/l)	67.00 (56.00, 80.00)	66.00 (55.00, 80.00)	70.00 (58.63, 82.25)	0.047*
Platelet count (× 10^9^/l)	190.00 (138.00, 242.00)	193.00 (142.25, 244.50)	177.20 (115.40, 232.00)	0.043*
Prothrombin time (s)	13.10 (12.60, 13.80)	13.00 (12.60, 13.70)	13.25 (12.59, 14.06)	0.158
Postoperative laboratory data				
AST (U/l)	101.00 (44.00, 209.00)	97.50 (45.00, 211.00)	110.50 (41.00, 208.00)	0.899
ALT (U/l)	84.50 (37.00, 188.25)	87.00 (37.00, 186.75)	73.33 (30.00, 196.67)	0.531
Alkaline phosphatase (U/l)	96.00 (67.00, 162.75)	97.50 (69.00, 170.00)	92.33 (63.75, 140.00)	0.145
Albumin (g/l)	33.10 (29.60, 36.13)	33.10 (29.93, 36.08)	33.04 (28.70, 36.40)	0.520
BUN (mmol/l)	4.38 (3.41, 5.44)	4.26 (3.37, 5.27)	4.66 (3.63, 5.65)	0.014*
Creatinine (μmol/l)	66.50 (55.00, 80.00)	66.00 (54.00, 79.75)	67.00 (56.70, 82.89)	0.111

*P value < 0.05 statistically significant, whether it is a high PCA sufentanil dose comparison between the two groups; Mann-Whitney U test; Chi-square test; n(%) = number (percentage); *PCA-H* High PCA sufentanil dosage, *PCA-NH* Non high PCA sufentanil dosage

The results related to tumor factors were obtained from pathological reports. The median tumor size was 3.2 cm (range, 0.3–18 cm). There were 71 cases of multiple tumors (12.6%), 138 cases of poor differentiation (24.6%), 60 cases of vascular infiltration (10.7%) and 116 cases of lymph node infiltration (20.6%). Table 4 shows a comparison of tumor factors between the two groups. There were no statistically significant differences in tumor factors between the two groups, including the tumor number, tumor size, cut margin, differentiation, vascular invasion and lymph node infiltration.

**Table 4:** Comparison of tumor factors between the PCA-NH group and the PCA-H group

	PCA-NH (n = 428)		PCA-H (n = 134)		*P*-value
	n (%)	Median (IQR)	n (%)	Median (IQR)	
Tumor number					
Solitary	371 (75.6)		120 (24.4)		0.383
Multiple	57 (80.3)		14 (19.7)		
Tumor’s size (cm)		3.20 (2.00, 5.00)		3.05 (2.04, 4.60)	0.522
Cut margin					
Free	407 (76.5)		125 (23.5)		0.416
Infiltrated	21 (70.0)		9 (30.0)		
Differentiation					
Well or moderate	323 (77.3)		95 (22.7)		0.142
Poor	100 (72.5)		38 (27.5)		
Vascular invasion					
No	386 (76.9)		116 (23.1)		0.236
Yes	42 (70.0)		18 (30.0)		
lymph nodes infiltration					
No	342 (76.7)		104 (23.3)		0.567
Yes	86 (74.1)		30 (25.9)		

*P value < 0.05 statistically significant; Mann-Whitney U test; Chi-square test; n (%) = number (percentage).

*PCA-H* High PCA sufentanil dosage, *PCA-NH* Non high PCA sufentanil dosage

### Sufentanil consumption 48 h after hepatectomy

The dosage of sufentanil at 48 h after hepatectomy varied widely, with the quartile range of consumption ranging from 64 to 133 μg. The median sufentanil consumption on the second day after surgery was less than twice as high as that on the first day, which may have been associated with less pain on the second day after surgery and less need for opioids.

After grouping the different factors, the amount of sufentanil at 48 h postoperatively was compared between the groups by the Mann-Whitney U-test, and it was found that the consumption of sufentanil was higher in cases of age ≤ 60 years (*P* < 0.001), major hepatic resection (*P* < 0.001), open approach (*P* < 0.001), operation time > 300 min (*P* = 0.008), intraoperative transfusion (*P* = 0.002) and PONV (*P* = 0.030). Sex, BMI, ASA classification, intraoperative blood loss, intraoperative infusion of RBCs, intraoperative dexmedetomidine use, postoperative NSAID use, and other indicators showed no significant differences between the groups (*P* > 0.05) (Table 5).

**Table 5:** Univariate analysis of influence on sufentanil consumption 48 hours after hepatectomy

Characteristics	Number (n, %)	Median, IQR	*P*
Age (years old)			
≤60	308 (54.8%)	98.00 (70.00, 143.50)	
>60	254 (45.2%)	82.00 (62.75, 120.00)	<0.001*
Gender			
Male	316 (56.2%)	94.50 (65.25, 139.00)	
Female	246 (43.8%)	88.00 (63.75, 124.00)	0.183
BMI (kg/m^2^)			
≤25	413 (73.5%)	90.00 (65.50, 131.50)	
>25	149 (26.5%)	92.00 (64.00, 134.00)	0.971
Intraoperative blood loss (ml)			
≤800	487 (86.7%)	90.00 (64.00, 131.00)	
>800	75 (13.3%)	99.00 (73.00, 155.00)	0.054
Operative time (min)			
≤300	422 (75.1%)	86.50 (63.00, 130.00)	
>300	140 (24.9%)	103.0 (71.25, 140.00)	0.008*
Intraoperative transfusion			
No	364 (64.8%)	86.50 (63.00, 125.00)	
Yes	198 (35.2%)	100.50 (71.50, 141.25)	0.002*
Intraoperative transfusion of RBC (u)			
≤2	453 (80.6%)	90.00 (64.00, 132.00)	
>2	109 (19.4%)	94.00 (72.50, 139.00)	0.165
Extent of resection			
Minor hepatic resection	272 (48.4%)	77.00 (57.00, 102.00)	
Major hepatic resection	290 (51.6%)	114.00 (76.00, 155.00)	<0.001*
Surgical approach			
Laparoscope	302 (53.7%)	82.00 (58.75, 119.25)	
Open	260 (46.3%)	106.50 (74.00, 147.75)	<0.001*
ASA classification			
I+II	506 (90.0%)	91.25 (64.88, 133.00)	
III	56 (10.0%)	86.00 (63.25, 135.00)	0.404
Introperative dexmedetomidine			
No	67 (11.9%)	91.50 (65.00, 133.00)	
Yes	495 (88.1%)	88.00 (62.00, 133.00)	0.758
Postoperative NSAIDs			
No	170 (30.3%)	92.00 (64.13, 132.00)	
Yes	392 (69.7%)	87.50 (64.00, 133.88)	0.848
PONV			
No	525 (93.4%)	90.00 (64.00, 130.50)	
Yes	37 (6.6%)	111.00 (76.50, 145.50)	0.030*
Tumor number			
Solitary	491(87.4%)	92.00 (64.00, 133.00)	
Multiple	71 (12.6%)	96.50(69.75, 141.25)	0.631
Tumor's size (cm)			
≤5	408 (72.6%)	92.00 (65.25, 133.75)	
>5	154 (27.4%)	86.50 (63.00, 127.00)	0.491
Cut margin			
Free	532 (94.7%)	90.00(64.25, 132.00)	
Infiltrated	30 (5.3%)	100.00 (63.75, 138.75)	0.499
Differentiation			
Well or moderate	424 (75.4%)	90.00 (64.00, 130.00)	
Poor	138 (24.6%)	92.00 (66.00, 140.00)	0.964
Vascular invasion			
No	502 (89.3%)	90.50 (64.00, 131.25)	
Yes	60 (10.7%)	94.50 (65.75, 146.50)	0.438
lymph nodes infiltration			
No	446(79.4%)	74.00(56.50, 112.00)	
Yes	116(20.6%)	97.00(64.75, 134.75)	0.540
Preoperative laboratory data			
AST (U/l)			
≤40	376 (66.9%)	93.50 (64.00, 135.50)	
>40	186 (33.1%)	90.00 (65.75, 128.25)	0.355
ALT (U/l)			
≤40	409 (72.8%)	94.00 (64.00, 134.50)	
>40	153 (27.2%)	85.00 (65.50, 125.00)	0.135
Alkaline phosphatase (U/l)			
≤94	211 (37.5%)	94.00 (62.00, 134.00)	
>94	351 (62.5%)	90.00 (67.00,132.00)	0.790
Albumin (g/l)			
≤35	155 (27.6%)	83.00 (63.00, 125.00)	
>35	407 (72.4%)	94.00 (65.00, 134.00)	0.112
BUN (mmol/l)			
≤7.5	522 (92.9%)	90.00 (64.00, 130.00)	
>7.5	40 (7.1%)	111.50 (72.00, 151.75)	0.072
Creatinine (μmol/l)			
≤73	362 (64.4%)	88.50 (65.00, 129.75)	
>73	200 (35.6%)	95.00(64.00, 139.00)	0.218
Platelet count (**×**10^9^/l)			
≤100	77 (13.7%)	97.00 (67.00, 139.00)	
>100	485 (86.3%)	90.00 (64.00, 131.50)	0.249
Prothrombin time (s)			
≤14	444 (79.0%)	90.50 (64.00, 130.00)	
>14	118 (21.0%)	93.50 (64.75, 137.00)	0.515
Postoperative laboratory data			
AST (U/l)			
≤40	121 (21.5%)	98.00 (70.00, 143.00)	
>40	441 (78.5%)	89.00 (64.00, 130.00)	0.162
ALT (U/l)			
≤40	159 (28.3%)	97.00 (72.00, 134.00)	
>40	403 (71.7%)	88.00 (63.00,132.00)	0.119
Alkaline phosphatase (U/l)			
≤94	252 (44.8%)	92.50 (64.00, 134.00)	
>94	310 (55.2%)	90.00 (66.00, 132.00)	0.775
Albumin (g/l)			
≤35	389 (69.2%)	88.00 (63.00, 131.00)	
>35	173 (30.8%)	97.00 (70,00, 133.50)	0.093
BUN (mmol/l)			
≤7.5	536 (95.4%)	90.00 (64.00, 132.00)	
>7.5	26 (4.6%)	118.50 (74.75, 177.25)	0.051
Creatinine (μmol/l)			
≤73	362 (64.4%)	90.00 (63.00, 133.00)	
>73	200 (35.6%)	95.00 (68.25, 132.75)	0.310

*P value <0.05 statistically significant; *PONV* Postoperative nausea and vomiting, *BUN* Blood urea nitrogen, *ALT* Alanine transaminase, *AST* glutamic oxalacetic transaminase.

### Correlation analysis of sufentanil consumption 48 h after surgery

In this study, as the variables did not conform to a normal distribution, Spearman correlation analysis was used to determine the relationship between sufentanil consumption 48 h postoperatively and other factors, including age, BMI, operation time, blood loss, intraoperative infusion of RBCs and FFP, pain at rest and during movement after surgery (day 1 and day 2), tumor size and laboratory data (including platelet count, prothrombin time, and indicators of liver and kidney function). The results showed that factors related to sufentanil consumption in the first 48 h after surgery were age, operation time, blood loss, intraoperative infusion of RBCs and FFP, pain during movement after surgery (day 1 and day 2), preoperative albumin and postoperative blood urea nitrogen (BUN), which showed a significant weak correlation (*P* < 0.05) (Table 6). Among them, age was negatively correlated with the increase in sufentanil consumption at 48 h after surgery; that is, as age decreased, the consumption gradually increased. Other factors were positively correlated with the increase in sufentanil consumption at 48 h after surgery; as these factors, including operation time, blood loss, intraoperative infusion of RBCs and FFP, movement pain after surgery (day 1 and day 2), preoperative albumin and postoperative BUN, increased, the consumption gradually increased.

**Table 6:** Correlation analysis of PCA sufentanil consumption in the first 48 h after surgery

		Age	Operation time	Blood loss	RBC	FFP	Move pain POD1	Move pain POD2	Pre Albumin	Post BUN
Sufentanil consumption	r	−0.192	0.166	0.185	0.086	0.106	0.099	0.094	0.100	0.084
	*P*	< 0.001	< 0.001	< 0.001	0.041	0.012	0.019	0.025	0.018	0.047

*Pre albumin* Preoperative albumin, *post BUN* Postoperative blood urea nitrogen, *POD1* Postoperative first day, *POD2* Postoperative second day

### Risk factors for sufentanil use 48 h after hepatectomy

The variables included in the multivariate analysis were based on the results of the univariate and correlation analyses of sufentanil consumption 48 h after hepatectomy. These variables included age, age group (≤ 60 and > 60 years), operation time, operation time group (≤300 and > 300 min), extent of resection (minor hepatic resection and major hepatic resection), surgical approach (laparoscope and open), blood loss, intraoperative transfusion (yes/no), PONV (yes/no), preoperative albumin, and postoperative BUN. In this study, the above variables were included in the binary logistic regression analysis to evaluate their influence on sufentanil consumption at 48 h after surgery. The obtained logistic model showed statistical significance (χ2 = 111.656, *P* < 0.001). Four variables were identified by the model, i.e., operation time (min), age group (≤ 60 and > 60 years), extent of resection (minor hepatic resection and major hepatic resection) and surgical approach (laparoscope and open). The specific parameter results are shown in Table 7. It can be concluded that a longer operation, age ≤ 60 years, major hepatic resection and an open approach are risk factors for increased sufentanil consumption 48 h after hepatectomy.

**Table 7:** Multivariate logistic regression analysis of risk factors for high PCA sufentanil dosage

	B	SE	*P*-Value	Odds ratio	95% CI for EXP(B)	
					Lower	Upper
Operative time (min)	0.002	0.001	0.033	1.002	1.000	1.004
Age (group)	0.866	0.233	< 0.001	2.377	1.505	3.755
Extent of resection	3.981	0.542	< 0.001	53.587	18.527	154.988
Surgical approach	2.516	0.518	< 0.001	12.381	4.488	34.157
Constant	−9.211	1.210	< 0.001	< 0.001		

*B* Regression coefficient, *SE* Standard error, *CI* Confidence interval, *Age (group)* Two age groups (≤ 60 and > 60 years old)

## Discussion

There have been many studies on the risk factors for long-term postoperative opioid use [11–13], but the factors influencing short-term postoperative opioid consumption have not been well studied. To more accurately determine the postoperative opioid needs of patients, we selected patients who used IV-PCA sufentanil at least 48 h after surgery as the subjects for the study. Other administration methods, such as oral administration or single intravenous injection, do not necessarily reflect patients’ real demand for opioids after surgery.

We found that the dosage of opioids after hepatectomy varied considerably among individuals in APS ward rounds. It is generally believed that postoperative pain affects the use of opioids [14], but there are many influencing factors that deserve further exploration. In this study, we found that an age ≤ 60 years, major hepatic resection, an open approach and a prolonged operation were likely to increase sufentanil consumption 48 h after hepatectomy. Understanding the risk factors for postoperative opioid use will help identify high-risk patients early, allowing us to make the necessary interventions to manage postoperative pain effectively. For high-risk patients, we should conduct close postoperative pain monitoring and increase the use of opioids, or we should appropriately reduce the use of opioids after surgery to avoid wasting these drugs.

In this study, we found that the dosage of sufentanil 48 h after hepatectomy was related to age. The use of opioids after surgery was higher in younger patients than in those who were older. This finding is consistent with the results of most previous studies [15–17]. Glasson et al. found that patients aged 54 years or less were more likely to use high-dose opioid analgesics than patients aged 55 or greater [15]. Similarly, Yen et al. and Lin et al. found that patients younger than 60 years received higher doses of opioid analgesics than those aged over 60 [16, 17].

How age affects postoperative opioid dosage is mainly considered as follows: First, older patients are more sensitive to opioid analgesics than younger patients [18]. Second, pharmacokinetic changes with age, including decreased volume of distribution, decreased metabolic function, and decreased elimination rate [19, 20], result in increased accumulation of opioids in elderly patients and prolonged effect intensity and duration. In addition, Kulkarni et al. [21] believe that young patients may experience greater emotional distress in the context of diseases that require surgery. Compared with young patients, elderly patients may regard serious diseases as the expected pain of aging. Such emotional upheavals, accompanied by symptoms of anxiety and depression, may exacerbate the severity of pain or disrupt healthy behaviors that may reduce pain symptoms. These emotional factors can also have a significant impact on postoperative pain. In general, the correlation between age and postoperative opioid use is generally recognized.

In this study, we found that the surgical approach was an influential factor for postoperative sufentanil dosage, suggesting a reduction in opioid dosage after laparoscopic surgery, which is consistent with the conclusions of many previous studies [22–24]. Mala T et al. found that patients undergoing laparoscopic hepatectomy used opioids for an average of 1 day, while patients undergoing open surgery used opiates for an average of 5 days [22].

A low demand for opioid treatment after laparoscopic surgery is related to a low degree of postoperative pain stress [25]. Since the incisions made in laparoscopic hepatectomy are small and only 4–5 holes need to be established, there is less pain stimulation [26–29]. Moreover, the local incision infiltration analgesia method can significantly reduce incisional pain and substantially reduce opioid use after surgery [30].

Additionally, the use of ultrasonic dissection in laparoscopic surgery significantly reduces smoke and eschar formation, fully ensuring that the surgeon has a clear visual field, and ultrasonic dissection does not damage the surrounding healthy tissues [31, 32]. Second, a harmonic scalpel, which is used to free the intrahepatic bile duct and blood vessels during laparoscopic surgery, can use electrocoagulation or clamping according to the thickness of the vessel for greater efficiency. Compared with tools in traditional open surgery, the use of a harmonic scalpel avoids the frequent replacement of other hand instruments and, at the same time, better guarantees electrocoagulation and hemostasis [31, 32]. These factors can significantly reduce pain stress and the need for opioids after surgery.

Studies have found that the extent of resection is an independent influencing factor for postoperative pain. Major hepatectomy is very traumatic, and most of it is performed through laparotomy [33]. During the operation, it is often necessary to use a liver retractor to pull the ribs, and sometimes it is necessary to remove the xiphoid process to fully expose the lesion [34]. Postoperative drainage tubes often lead to severe postoperative pain in patients. This is the main reason for the increased consumption of sufentanil after surgery.

This study found that the operation time was a risk factor for a high postoperative sufentanil dosage. Patients with operation times longer than 300 min consumed more sufentanil within 48 h after the operation, but few related studies have investigated this factor. Loriga B et al. [35] found that the operation time was a significant risk factor for postoperative pain after vitreoretinal surgery, and this conclusion was related to the choice of anesthesia. Local anesthesia can enhance this correlation because with increases in the operation time and decreases in the anesthetic effect, postoperative pain stimulation increases. Silins V et al. [36] found that the operation time was an independent predictor for increased morphine consumption in children three days after the operation. It is believed that the operation time might be related to invasiveness, reflecting the degree of activity. Indeed, the liver is rich in blood vessels and has a complex anatomy, and the long operation could reflect the degree of trauma. The longer the operation, the stronger the inflammatory response caused by the inflammatory factors released from the injury, the higher the pain stress, and the greater the demand for opioid drugs after the operation. Unfortunately, the perioperative levels of leukocytes and other inflammatory indices were not included in this study, which could have helped determine the relationship between the duration of surgery and postoperative sufentanil dosage.

Opioid metabolism and clearance are affected by liver and renal function, but in this study, no relationship between perioperative liver and renal function and postoperative sufentanil dosage was found. Sufentanil is metabolized to normethyl sufentanil in liver microsomes, and the activity of normethyl sufentanil is only 10% that of sufentanil [37]. Renal function has a weak effect on the pharmacokinetics of sufentanil [38], which may be the main reason for these findings; of course, this requires further studies with large samples and multiple centers.

Sex has not been identified in previous studies as an influential factor for postoperative analgesic dosage consistency [39]. Many research studies have reported that women are more likely than men to suffer postoperative pain and use more analgesic drugs [40, 41]. Zheng H et al. found that women used 25.8% more opioids than men within 24 h after surgery [41]. It is generally concluded in many studies that women tend to report higher levels of anxiety and to exhibit factors associated with pain [40, 41]. However, the current study is consistent with that conducted by Lin et al., in that no effect of sex on postoperative sufentanil consumption was found [17].

Similar to the study by Lee Y et al., the present study did not find that BMI was a risk factor for sufentanil dosage 48 h after surgery [14]. However, studies have shown that BMI is positively correlated with opioid consumption, but the etiology is still unclear [42, 43]. Kvarda P et al. considered that this finding might be related to the bias of surgeons against the use of opioids in obese patients because surgeons believed that patients with higher BMIs metabolized opioids differently and therefore used more analgesics in obese patients [42]. Previous studies on the relationship between these factors and postoperative opioid dosage are few, and further prospective studies are needed.

Similarly, this study did not find that PONV was a factor influencing the postoperative sufentanil dosage, although the postoperative sufentanil dosage was higher in the PONV group. The incidence of nausea and vomiting, in addition to opioid use, would also be affected by many factors, including the type of patient, surgery, and anesthesia [44, 45].

A recent meta-analysis of randomized controlled trials showed that dexmedetomidine saves opioids and can significantly reduce their dosage [46]. Studies have demonstrated that anti-inflammatory and analgesic drugs (e.g., NSAIDs), such as parexib or flurbiprofen, can reduce postoperative pain and reduce postoperative opioid use [47, 48]. However, no effect of intraoperative dexmedetomidine and postoperative NSAID use on the postoperative sufentanil dosage was found in this study. In our study, we also did not find an association between intraoperative blood loss, preoperative platelet count, prothrombin time, or tumor factors and the 48-h postoperative sufentanil dose. There have been few studies on the relationship between these factors and postoperative opioid dosage, and further prospective studies are needed.

This study has several limitations. The design and outcome reliability of retrospective studies is inherently dependent on the accuracy and completeness of the documents available in electronic medical records and surgical reports. The present sample included 562 patients after hepatectomy. As a result, the sample size may not be sufficient to identify the effects of individual variables. Therefore, it is suggested that a larger sample size be studied in the future. In addition, this was a retrospective study that did not confirm the difference between the doses of different opioid analgesics. Future studies should include treatment with other opioids. Postoperative pain and opioid consumption are also influenced by a patient’s psychological factors, the preoperative experience of pain, and the preoperative use of opioids [49–51]. It is difficult to evaluate these factors accurately in retrospective investigations; therefore, to comprehensively assess the factors affecting postoperative opioid dosage, it is necessary to add these predictive factors to future prospective studies.

Finally, patient education level and cognitive ability, postoperative infection, postoperative rehabilitation exercise, and other factors may be related to acute postoperative pain and opioid requirements, which can be further explored by adding corresponding observational indicators in future studies.

## Conclusions

This retrospective study was designed to determine the influencing factors of sufentanil consumption 48 h after hepatectomy. The results of this investigation can help anesthesiologists improve the quality of perioperative pain and opioid management by considering these influencing factors. The findings of this study suggest that the factors influencing sufentanil consumption in liver cancer patients after hepatectomy are the age group (≤ 60 and > 60 years), extent of resection (minor hepatic resection and major hepatic resection), surgical approach (laparoscope and open) and operation time (min). Patients younger than 60 years, patients who underwent major hepatic resection, patients who underwent surgery with an open approach and patients who had a longer operation tended to consume more sufentanil after surgery. These findings have a certain value for guiding the management of pain during the perioperative period in liver resection.

## Data Availability

The datasets used and/or analyzed during the current study are available from the corresponding author on reasonable request.

## Abbreviations

BMI: Body mass index
ASA: American Society of Anesthesiologists
PCA: Patient-controlled analgesia
IV-PCA: Intravenous patient-controlled analgesia
NRS: Numerical rating scale
APS: Acute Pain Service Team
DEX: Dexmedetomidine
NSAIDs: Anti-inflammatory and analgesic drugs
POD: Postoperative day
RBC: Red blood cells
FFP: Fresh frozen plasma
PONV: Postoperative nausea and vomiting
BUN: Blood urea nitrogen
ALT: Alanine transaminase
AST: Glutamic oxalacetic transaminase
